# Immediate implant placement in the anterior mandible: a cone beam computed tomography study

**DOI:** 10.1186/s12903-024-04111-1

**Published:** 2024-03-27

**Authors:** Emmy Kanewoff, Reem Alhallak, Vinícius de Carvalho Machado, Bruno Ramos Chrcanovic

**Affiliations:** 1 Undergraduate student, Faculty of Odontology, Malmö University, Malmö, Sweden; 2 Slice Diagnóstico Volumétrico por Imagem, Belo Horizonte, Brazil; 3 Department of Oral and Maxillofacial Surgery and Oral Medicine, Faculty of Odontology, Malmö University, Malmö, Sweden

**Keywords:** Dental implants, Immediate implant placement, Cone beam computed tomography, Virtual planning, Anterior mandible.

## Abstract

**Background:**

The placement of implants into the alveolar socket right after tooth extraction is called immediate implant placement (IIP). This approach has its particularities depending on which region of the jaws is involved. The anterior mandible region is peculiar due to the presence of mandibular incisors, which have the shortest roots among all permanent teeth.

**Purpose:**

This study aimed to investigate the factors that could be associated with the risk of either cortical bone wall perforation or invasion of the 2 mm secure distance from the surrounding anatomical structures (defined as unsafe implant placement), with IIP in the maxillary aesthetic zone, in a cone-beam computed tomography (CBCT) virtual study.

**Materials and methods:**

CBCT exams from 239 eligible subjects were investigated. Implants were virtually placed in two distinct positions: prosthetically-driven (along the long axis of the existing tooth) and bone-driven position (according to the available bone and with regard to nearby anatomical structures). Correlation between several variables was tested, and binary logistic regression analysis in order to assess of the possible associations between covariates and unsafe placement was performed.

**Results:**

Safe placing implants was significantly higher for the bone-driven in comparison to the prosthetically-driven position (22.2% vs. 3.3%, respectively), and the 2-mm secure distance from anatomical structures was not possible to respect in the majority of cases (77.6% vs. 82.9%, respectively). Covariates associated with a higher risk of unsafe placement were tooth region (CI in relation to IL and CA), decrease of labial concavity angle (LCA), decrease of mandible basal bone height (MBBH), and decrease in mandibular bone thickness at the tooth apex level (MBT0).

**Conclusion:**

The possibility of safely placing immediate implants in the anterior mandible is significantly higher for bone-driven than in prosthetically driven position. Presurgical virtual planning with CBCT is a great tool for minimizing the risk of implant unsafe placement with regards to the anatomical conditions in the mandible.

## Introduction

It was initially recommended that a dental implant should be surgically placed in the planned surgical site only after a period of months of healing of the alveolar socket after tooth extraction, so adequate remodeling and healing of the alveolar bone would occur in order to optimize osseointegration of the implant [1]. Not so long time thereafter the placement of implants into the alveolar socket right after tooth extraction was proposed [2], or the immediate implant placement (IIP) approach.

IIP has several advantages in comparison to the initial protocol, such as reduction in treatment time, decrease in the number of surgical sessions, decrease of the alveolar bone post-extraction resorption, positive psychological impact on the patient, and possible ability to place the implant in an ideal axial position in relation to the tooth that once occupied the socket [3]. However, the approach may decrease the chance of primary stability of the implant, due to the reduced amount of bone, as the alveolar socket is empty [4].

The immediate placement of implants is a viable option even when placed in alveolar sockets of teeth presenting endodontic and periodontal lesions, provided that proper care is taken [5]. The findings of a systematic review on the subject, gathering together data from 163 studies, showed a failure rate of 3.60% (622 failures out of 17,278 implants) implants placed in fresh extraction sockets in comparison to 2.87% (1,113 out of 38,738 implants) for implants placed in healed sites, although the meta-analysis showed that there is a higher risk of failure for implants placed in extraction sockets [6].

The IIP approach has its particularities depending on which region of the jaws is involved. The anterior mandible region is peculiar due to the presence of mandibular incisors, which have the shortest roots among all permanent teeth [7]. Having that in mind, it is important that an adequate pre-treatment evaluation is conducted in the cases which IIP is planned [8].

The aim of the present study was to investigate the factors that could be associated with the risk of either perforation of cortical bone plates or invasion of the secure distance from the surrounding anatomical structures with IIP in the anterior mandibular area, in a cone-beam computed tomography (CBCT) virtual study. The null hypothesis of the present study was that there will not be a significant difference in the prevalence of either cortical bone perforation or of invasion of the 2 mm secure distance from the surrounding anatomical structures (unsafe implant placement) between bone-driven and prosthetically-driven ideal position for IIP in the anterior mandible, against the alternative hypothesis of a difference.

## Materials and methods

The methodology of the present study is, to a great extent, similar to the one adopted in a previous already published study [9], but focusing on the anterior mandible instead of on the anterior maxilla.

### Specific objectives

The purposes of the present CBCT-scan virtual planning study were (1) to determine the risk of perforation of either the labial or lingual bone plate and of invasion of the 2 mm secure distance from these surrounding anatomical structures (defined as *unsafe implant placement*) when implants are virtually planned to be placed either along the longitudinal axis of the tooth in six anterior mandibular teeth areas (central incisor – CI, lateral incisor – LI, canine - CA) or in a bone-driven position, in case of immediate implant placement, (2) to determine the minimal implant length possible without perforation, when respecting a secure distance from adjacent anatomical structures, (3) determine the angle between the implants in the two aforementioned positions, and (4) to assess possible associations between all the covariates and cortical bone perforation when the implant is planned in the ideal bone-driven position [9].

### Subjects

The present retrospective analysis was based on the mandibular scans performed in private radiology company Slice Diagnóstico Volumétrico por Imagem, in the city of Belo Horizonte, Brazil, during the last quarter of the year 2014. The scans used in the present study were selected from the CBCT database and were not specifically acquired for this publication.

### Ethical considerations

The study was approved by the local Ethics Committee (PUC-MG, Belo Horizonte, Brazil Protocol code CAAE 0001.0.213.000–10). The patients were contacted through a telephone call and a signed informed and written consent form was obtained from each patient approving the use of their scans. The patients were not identifiable in any way, and a decoding list linking patient names and numbers was used and stored by the principal investigator, which was destroyed after completion of the study. The investigation was conducted according to the principles embodied in the Helsinki Declaration of 1964 for biomedical research involving human subjects, as amended in 2013.

### Inclusion and exclusion criteria

The following inclusion criteria were applied: (a) CBCT examinations from patients who allowed use of their scans; (b) CBCT examinations of the mandible; (c) presence of fully erupted anterior mandibular teeth; (d) each tooth had to have fully formed apexes; and (e) each tooth had to be normally positioned and have normal alignment.

CBCT examinations were excluded on the basis of (a) the presence of technical artifacts that hindered the evaluation of the focused structures, (b) images had an implant, a pathologic lesion, evident root resorption, or a missing tooth, and (c) examinations from patients that had a history of orthognathic surgery, grafted alveolar ridge, supernumerary or impacted teeth, preexisting alveolar bone destruction, perforation, dehiscence, or a combination of these caused by periodontal disease or traumatic injury around the investigated region [9].

### Hardware and software

The methodology was basically the same as adopted in other study [9], but for the mandible. CBCT scanning was performed with an i-CAT CBCT system (Imaging Sciences International, Hatfield, PA, USA). The scans were acquired using the i-CAT 3D Imaging System (i-CAT Vision Software, Imaging Sciences International, Hatfield, PA, USA) and included the entire mandible. The following CBCT scan parameters were used for all patients: a tube voltage of 110 kV, 1 to 20 mA, emission of x-rays over an interval of 40 s, and an effective dose of 136 µSV. Measurements were obtained on the transversal sections of the selected teeth, with the use of a computer software (DeltalSlice Navegação Virtual, Bioparts, version 2021, Brasília, Brazil). The distance between the obtained transversal sections were 1.0 mm. The field of view (FOV) was standard (medium; 6 × 14 cm), capturing the entire mandible.

### Sample size calculation

The calculation, performed with ClinCalc.com, was based on the study of Botermans et al. [9], in which an incidence of about 5% of fenestration (cortical bone perforation) for IIP in bone-driven ideal position planned for the anterior maxilla was observed. We hypothesized that IIP placed in the anterior mandibular sockets in the prosthetically-driven would result in four times as much cortical bone perforation in comparison to IIP placed in a bone-driven ideal position, namely 20% vs. 5%. There was a need of 150 cases in total, setting alpha at 5% and power at 80%.

### Calibration

Two researchers (E.K. and R.A.) were calibrated using 10 randomly selected CBCT exams. The reproducibility of measurements among observers measuring the same quantity to one-tenth of a millimeter was calculated at a correlation of 0.95 for the 10 scans.

### Definitions and measurements

Secure distance from implant to the adjacent anatomical structures. Implants were placed according to a secure distance from adjacent anatomical structures. In the anterior mandible these were adjacent teeth, and labial and lingual cortical bone plates. Distance between the implant and the structures was defined as the distance between the closest point of the implant to the aforementioned structures. The minimum distance between an implant and the adjacent tooth was established as 2 mm, according to the recommendations that this distance should not be shorter than 1.5 to 2 mm [10]. Moreover, a 2-mm secure distance was kept from all external cortical bone plates [9].

Implant simulation (based on [9]). The center of the implant platform was positioned along an imaginary line along the long axis of the tooth, in buccolingual sections of the exams. A parallel implant was selected for virtual IIP. The implants chosen for the sockets were 3.0 mm in diameter for CI and LI, and 3.75 mm in diameter for CA.

For all the simulated implants, the implant platform was positioned 1 mm below the buccal crestal level, in order to follow the approximated 3-year mean marginal bone loss for immediately implants placed [11]. Moreover, the minimal amount of bone apical to the alveolar socket apex required to achieve primary stability has been considered to be 4 mm to minimize the risk of early implant loss [12, 13].

**Fig. 1:**
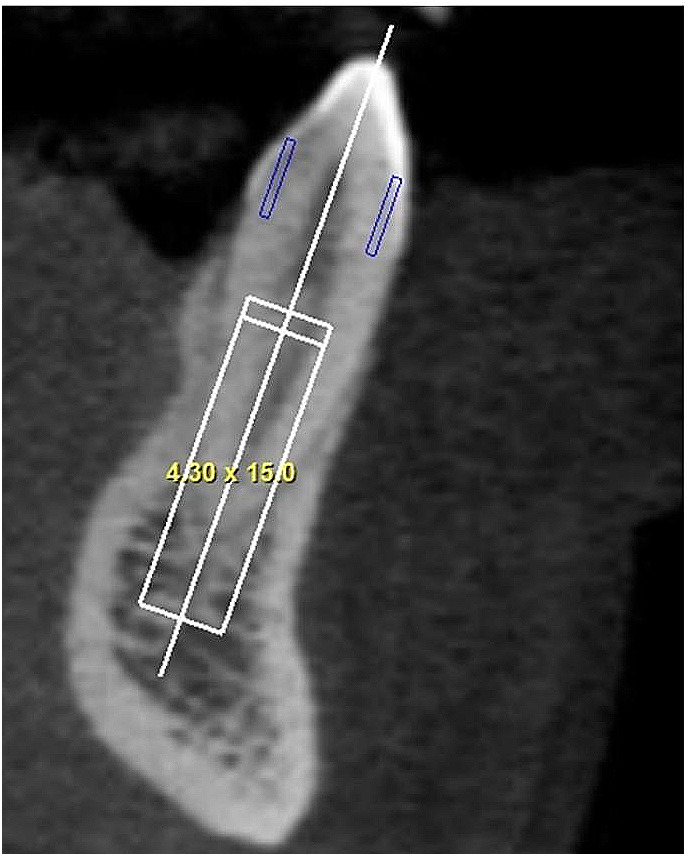
Absence of bone plate perforation, when implants are virtually planned in the prosthetically-driven ideal position, respecting the minimum of 4-mm of apical anchorage

**Fig. 2:**
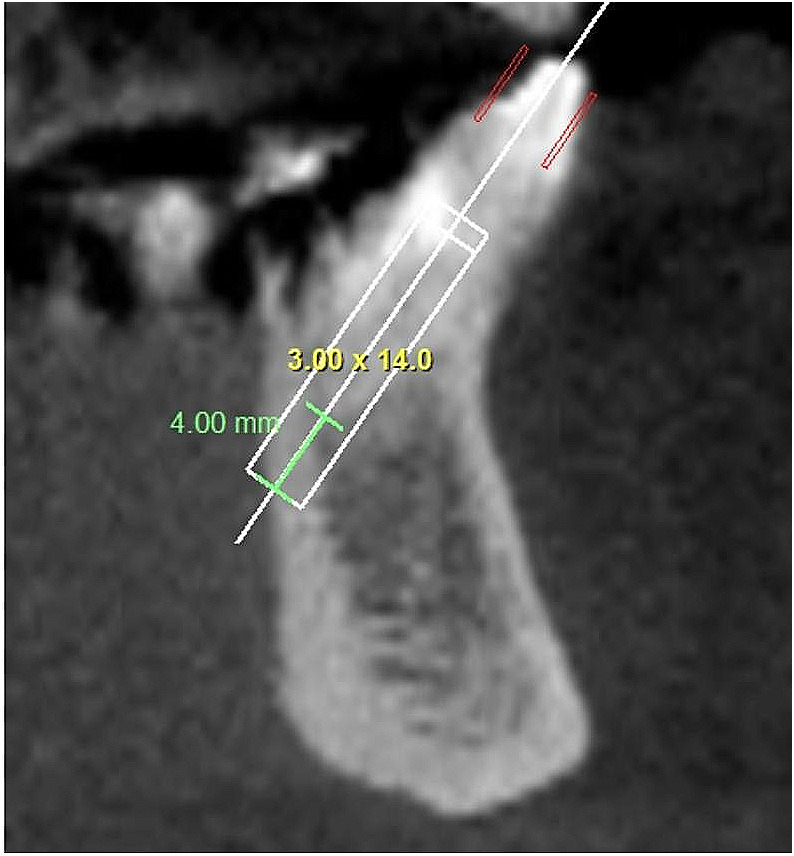
Occurrence of bone plate perforation, when implants are virtually planned in the prosthetically-driven ideal position, respecting the minimum of 4-mm of apical anchorage

In each tooth site, implants were positioned in two ways:

(a) Prosthetically-driven ideal position: Implant placed along or parallel to line A (which was defined as the line connecting the incisal border and the root apex of the tooth, bisecting the labial and lingual halves of the tooth), with at least 4 mm of native bone in ideal position according to the position of the real tooth crown shown in the sagittal section. Depending on the case, this could lead to absence (Fig. 1) or occurrence (Fig. 2) of bone plate perforation. In absence of perforation, it was also noted if the implant respected the 2 mm distance to adjacent anatomical structures. The proper mesio-distal angulation was also verified in the panoramic view [9];

(b) Bone-driven ideal position: Defined as the placement of the implant without perforation, when anchoring the implant apex with 4 mm of native bone, still respecting the minimum 2 mm distance away from the from the external labial and lingual bone plates. The proper angulation was also verified mesio-distally in the panoramic view of the CBCT exam [9].

**Fig. 3:**
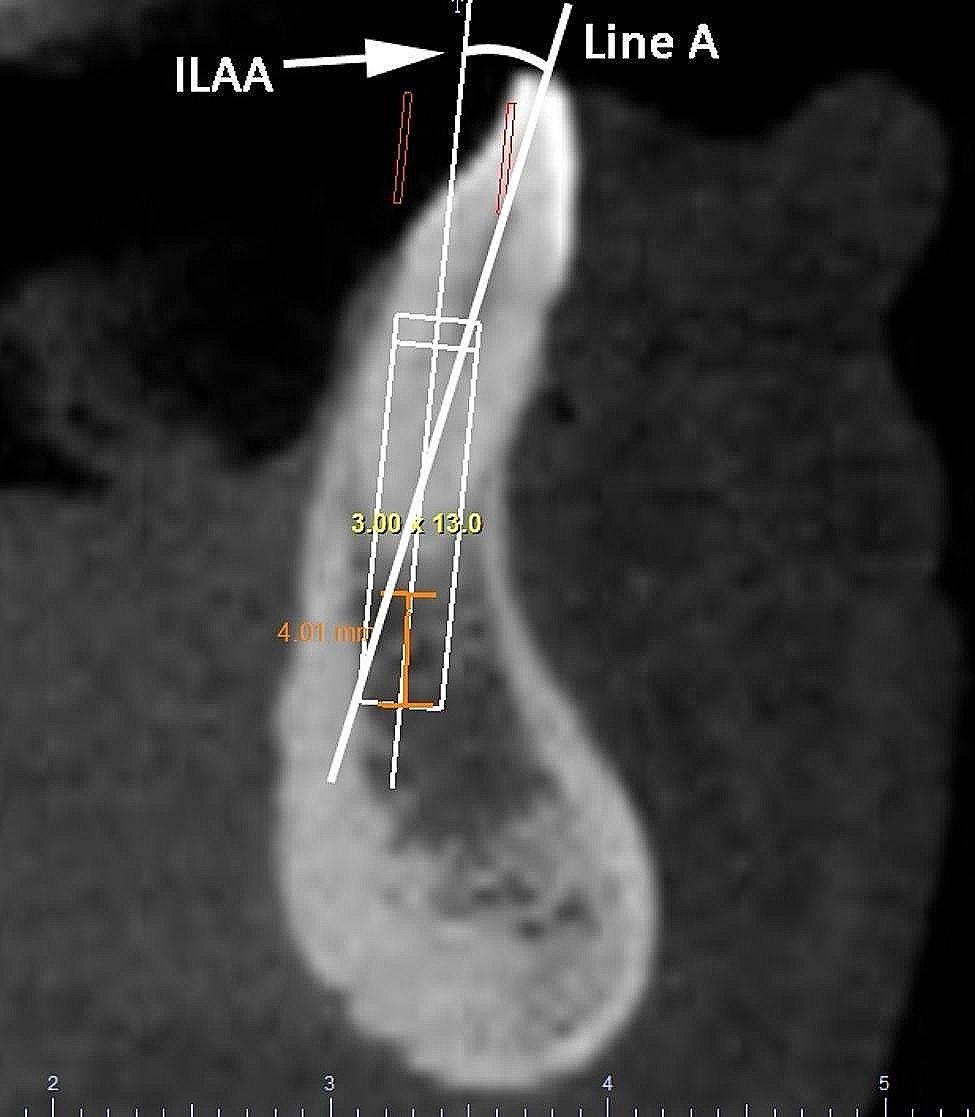
The implant-line A angle (ILAA)

Implant-line A angle (ILAA) (Fig. 3). The angle of the implant in this position in relation to the position of the prosthetically-driven ideal position (line A) was determined, the implant-line A angle (ILAA), in buccolingual sections of the exams [9].

**Fig. 4:**
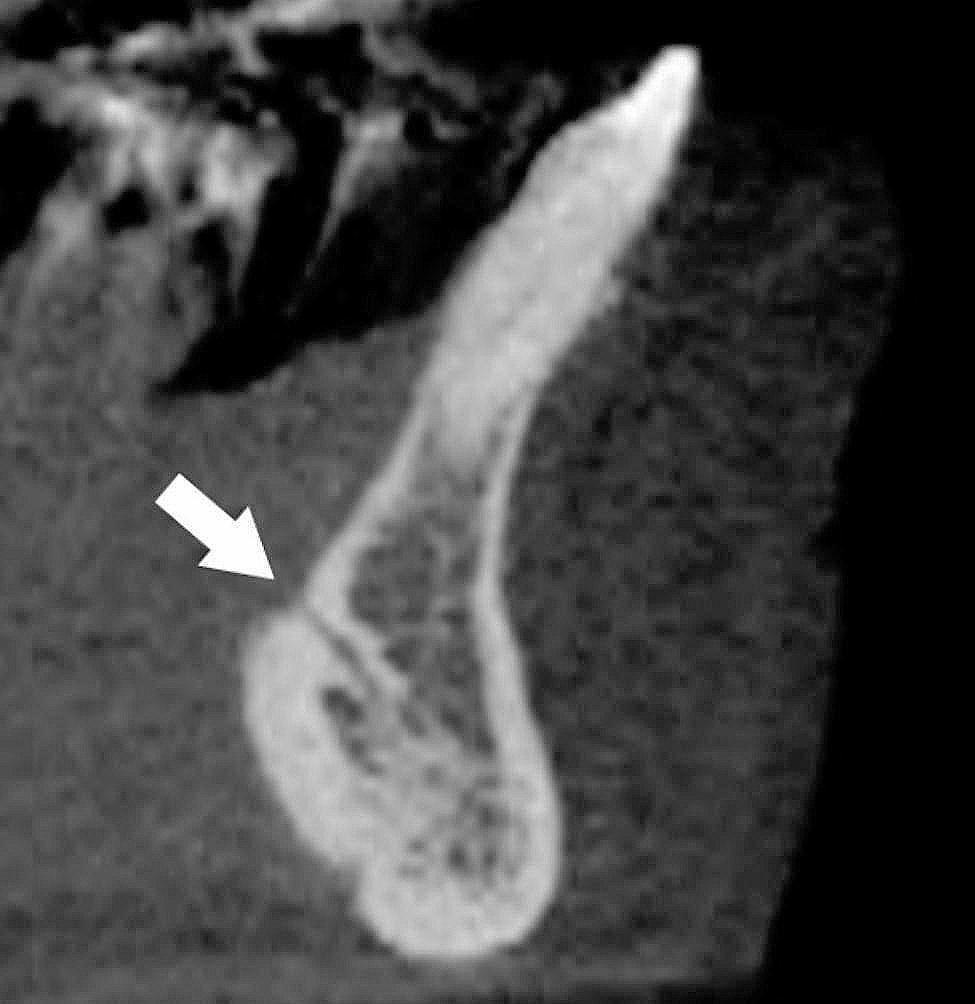
A mandibular lingual foramen (arrow)

Position of the mandibular lingual foramen (Fig. 4). The number and location of the lingual foramen (or foramina) in relation to mandibular teeth was verified, in buccolingual sections of the CBCT exams. The lingual foramen contains an artery that develops from the anastomosis of the two sublingual arteries, and is usually situated in the midline of the internal region of the mandibular symphysis at the level, or superior to the mental spines. There may be two or more foramens at the mandibular midline and their location and dimensions are quite variable [14].

**Fig. 5:**
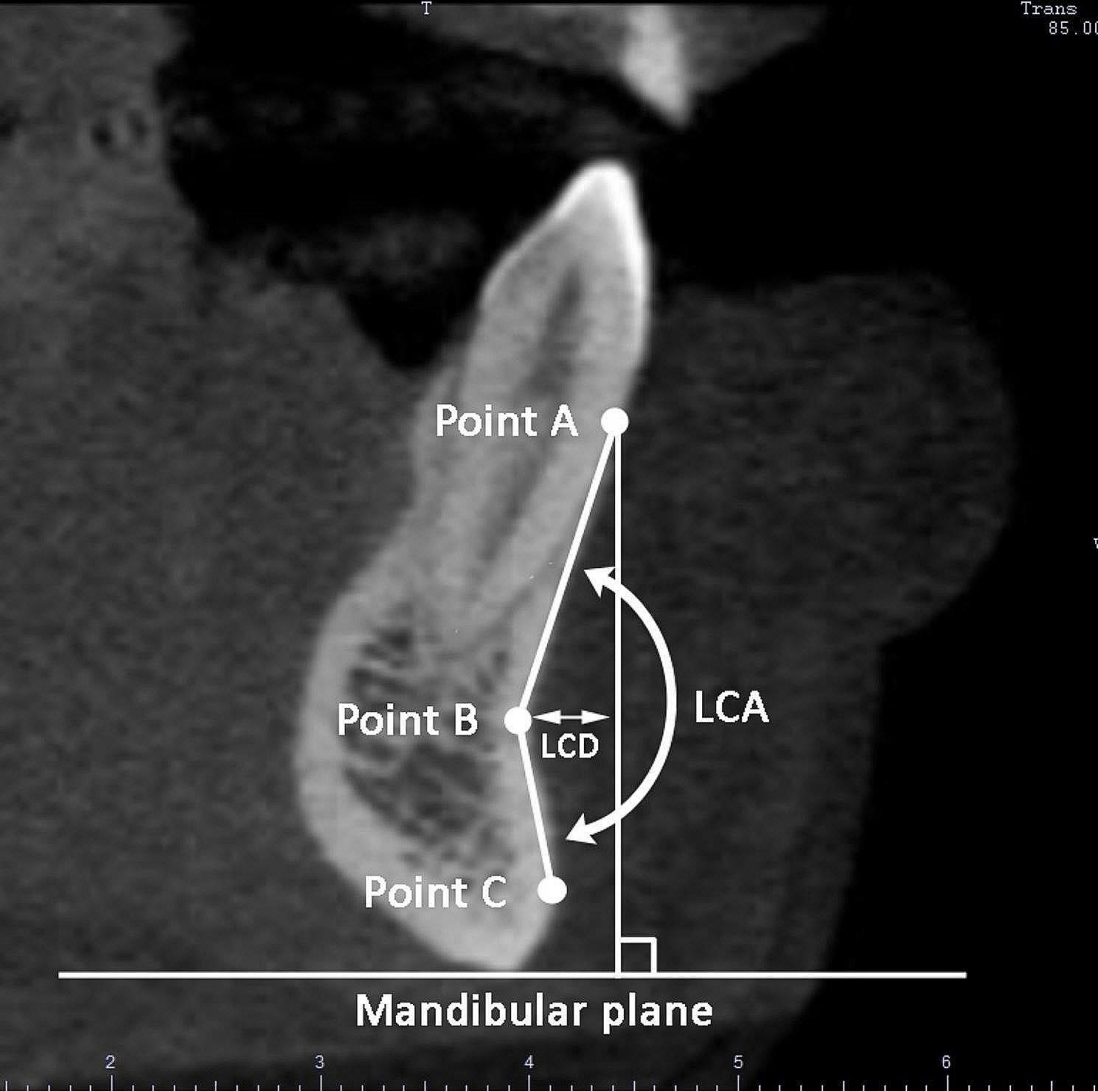
Labial concavity angle (LCA) and labial concavity depth (LCD)

Labial concavity angle (LCA) (Fig. 5). The LCA was defined as the angle between the line A-B and the line B-C. Line A-B: the line connecting points A and B, with point A defined as the most external (anterior) point of the labial plate close to the cemento-enamel junction, and point B as the deepest (most posterior) point in the bony labial outline. Line B-C: the line connecting points B and C, with point C defined as the most external (anterior) point of the labial plate inferior to point B. The LCA was measured at the virtual buccal-lingual longitudinal middle section of each tooth [9].

Labial concavity depth (LCD) (Fig. 5). The distance between the deepest point of the labial bone plate (point B) and a vertical reference line perpendicular to the mandibular plane, passing through the most external point of the labial plate (point A) [15].

**Fig. 6:**
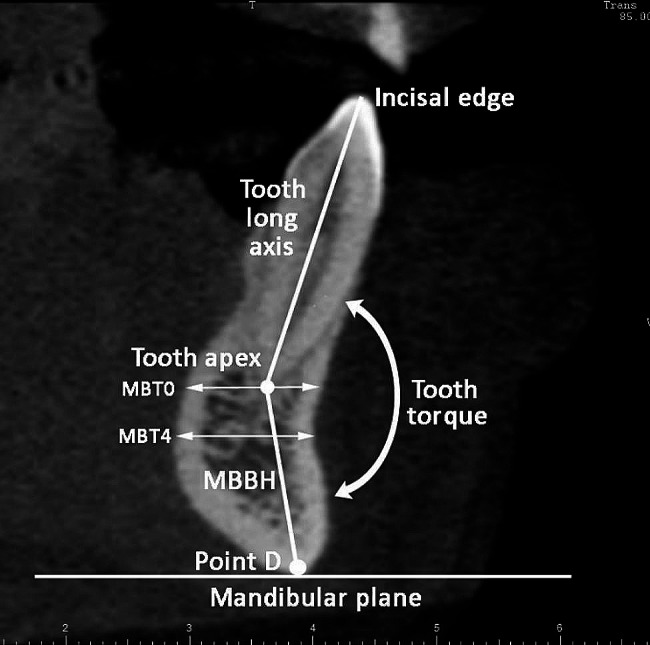
Mandible basal bone height (MBBH), tooth torque (TT), and mandibular bone thickness (MBT0, at tooth apex; MBT4, at 4 mm inferior to MBL0)

Mandible basal bone height (MBBH) (Fig. 6). Measured from the tooth apex to the lowest point on the bony outline of the mandible (point D) [16].

Tooth torque (TT) (Fig. 6). The angle formed between the long axis of a tooth (the line connecting incisal edge and root apex of the tooth) and MBBH [16].

Mandibular bone thickness (MBT) (Fig. 6). The mandibular labial-lingual bone thickness was measured at the tooth root apex level (MBT0) and at 4 mm inferior from it (MBT4). Each measurement line was parallel to the mandibular plane.

Angle measurement. The images generated were later transferred to the Image J software (National Institute of Health, Bethesda, USA) in order to measure the angles involved in the study.

### Statistical analyses

The statistical analyses adopted in the present study were, to a great extent, similar to the ones adopted in a previous already published study [9].

The mean, standard deviation, minimum and maximum for each of the measurements were calculated. Variations were evaluated according to the tooth (CI, LI, CA), the predictor variable. The other variables were the mandibular side (left/right), age, and sex. Kolmogorov–Smirnov test was performed to evaluate the normal distribution. Levene test evaluated homoscedasticity. Paired t-test and Wilcoxon test, where indicated, were performed to compare the measurements of each tooth between the left and right side of the mandible. The performed tests for the comparison of independent groups (tooth, sex) were Student’s *t*-test or Mann-Whitney test, depending on the normality. Pearson’s chi-squared test or Fisher’s exact test were used for categorical variables, depending on the expected count of events in a 2 × 2 contingency table. Pearson correlation and linear regression were performed to verify the relationship between several variables, namely the patients’ age, ILAA, LCA, LCD, MBBH, TT, MBT0, MBT4, and the minimal implant length possible. Spearman correlation was performed to check the relationship among the categorical variables, and between these and the and continuous variables.

Univariate and multivariable binary logistic regressions were used to assess possible associations between all the covariates and unsafe placement when the implants were planned in the bone-driven ideal position. Odds ratio (OD) and 95% confidence intervals (95% CI) were estimated from the regression models.

For the final multivariable regression model, only the variables that were moderately associated (*p* < 0.10) with unsafe placement and did not present multicollinearity were included. In order to verify multicollinearity, a correlation matrix of all of the predictor variables with a significant OD (*p* value cut-off point of 0.1) identified in the univariate models was scanned, to see whether there were some high correlations among the predictors. Collinearity statistics obtaining variance inflation factor (VIF) and tolerance statistic were also performed to detect more subtle forms of multicollinearity.

The degree of statistical significance was considered *p* < 0.05. These data were statistically analyzed using the SPSS version 28 software (IBM Corp., Chicago, IL, USA).

## Results

### Selection of cases

From the 475 CBCT exams of the mandible performed at the aforementioned oral radiology company during the last quarter of 2014, 236 exams were excluded either due to one or more missing teeth in the focused area, presence of an implant in the premolar area, presence of pathologies, presence of teeth with misalignment, or due to the presence of radiological artefacts that hindered the evaluation of the focused structures. The remaining 239 CBCT exams were included in the study.

### Description of the cohort group

The description of the cohort group is shown in Table 1. There were more exams from women than from men, and the mean age was similar between men and women.

**Table 1:** Description of the cohort group, according to sex

Individuals (n)	239
	*Individuals / teeth (n)*
Male	98 / 588
Female	141 / 846
	*Age, mean ± SD (min, max) (years)*
Male	50.9 ± 15.6 (14.2, 86.3)
Female	49.0 ± 14.3 (19.5, 89.0)

SD – standard deviation

### Measurements

The mean minimum length of the planned implants when in bone-driven position, without perforation or invasion of the 2 mm secure distance from the surrounding anatomical structures is shown in Table 2. The mean values were higher for CA than for IL, and the latter were higher than for IC. The difference of the mean values was statistically significant different between IC and IL (*p* < 0.001, Mann-Whitney test), IC and CA (*p* < 0.001, Mann-Whitney test), and between IL and CA *p* < 0.001, Mann-Whitney test).

**Table 2:** Minimum length of the implants when planned in bone-driven position

Tooth	mean ± SD (min, max) (n)
**43**	15.2 ± 2.0 (10.5, 21.0) (85)
**42**	13.3 ± 1.7 (10.0, 17.0) (56)
**41**	12.2 ± 2.2 (9.5, 16.5) (17)
**31**	11.9 ± 1.7 (10.0, 16.5) (16)
**32**	13.4 ± 1.7 (10.0, 18.5) (54)
**33**	15.7 ± 2.0 (11.0, 20.5) (82)
**Global**	14.3 ± 2.3 (9.5, 21.0) (310)

SD – standard deviation

Table 3 shows the values for the LCA according to different tooth positions, as well as for the different sexes. There was a statistically significant difference of LCA mean value between the groups of male and female patients, when all teeth were considered (*p* < 0.001, Mann-Whitney test), when all measurements were considered. There was a very weak correlation between LCA and sex of the individuals (*r*_s_ = -0.099, *p* < 0.001; Spearman correlation).

**Table 3:** Labial concavity angle (LCA) values, global and for the different sexes

Tooth	LCA - mean ± SD (min, max)			p value *
	Global (*n* = 239 each tooth)	Male (*n* = 98 each tooth)	Female (*n* = 141 each tooth)	
**43**	145.7 ± 8.1 (120.4, 168.7)	146.5 ± 8.3 (123.4, 164.8)	145.2 ± 8.0 (120.4, 168.7)	0.185
**42**	143.9 ± 7.6 (121.0, 162.2)	144.7 ± 7.8 (121.0, 162.2)	143.4 ± 7.5 (121.0, 159.6)	0.202
**41**	145.6 ± 7.8 (123.5, 172.0)	146.7 ± 7.6 (128.8, 170.5)	144.9 ± 8.0 (123.5, 172.0)	0.059
**31**	145.0 ± 8.0 (121.6, 169.6)	146.1 ± 7.7 (126.7, 169.2)	144.3 ± 8.2 (121.6, 169.6)	0.069
**32**	144.1 ± 7.8 (121.5, 193.9)	145.3 ± 8.6 (129.2, 193.9)	143.3 ± 7.2 (121.5, 159.8)	0.180
**33**	145.8 ± 8.7 (114.4, 170.8)	146.4 ± 9.4 (114.4, 170.8)	145.4 ± 8.1 (125.8, 167.2)	0.190
**All teeth**	145.0 ± 8.1 (114.4, 193.9) (*n* = 1,434)	145.9 ± 8.2 (114.4, 193.9) (*n* = 588)	144.4 ± 7.9 (120.4, 172.0) (*n* = 846)	< 0.001

LCA - Labial concavity angle

SD – standard deviation

* Comparison of the LCA mean values between male and female individuals; Mann-Whitney test

Table 4 shows the frequency of cortical bone perforation for both prosthetically- and bone-driven positions, and the ILAA. It can be observed that the frequency of perforation is higher when the implants are planned in the prosthetically-driven position in relation to implants planned in the bone-driven positions. The difference of the prevalence of cortical bone perforation between prosthetically- and bone-driven ideal position was highly statistically significant (*p* < 0.001, Pearson’s chi-squared test). There were only 47 cases (out of 1,434) without perforation, for the prosthetically-driven implants. The mean ILAA angle was determined 10.5 ± 6.2 degrees. The mean ILAA was higher in CI than in IL and CA regions. There was a statistically significant difference for the mean ILAA values between IC and IL (*p* = 0.043, Mann-Whitney test), but not between IC and CA (*p* = 0.054, Mann-Whitney test), nor between IL and CA (*p* = 0.371, Mann-Whitney test).

**Table 4:** Frequency of cortical bone perforation, for both prosthetically- and bone-driven positions, and the implant-line A angle (ILAA)

Tooth	Prosthetically driven			ILAA	Bone driven		
	No perforation	< 2 mm	Perforation		No perforation	< 2 mm	Perforation
		n (%)		mean ± SD (min, max)		n (%)	
**43**	15 (6.3)	179 (74.9)	45 (18.8)	11.0 ± 6.7 (0.4, 25.6)	85 (35.6)	153 (64.0)	1 (0.4)
**42**	8 (3.3)	205 (85.8)	26 (10.9)	9.2 ± 5.8 (3.3, 24.8)	56 (23.4)	182 (76.2)	1 (0.4)
**41**	2 (0.8)	208 (87.0)	29 (12.1)	15.6 ± 8.8 (4.2, 26.8)	19 (7.9)	219 (91.6)	1 (0.4)
**31**	1 (0.4)	206 (86.2)	32 (13.4)	14.8 ± 8.6 (5.6, 24.9)	18 (7.5)	221 (92.5)	0 (0.0)
**32**	11 (4.6)	207 (86.6)	21 (8.8)	9.1 ± 3.9 (3.6, 20.4)	55 (23.0)	184 (77.0)	0 (0.0)
**33**	10 (4.2)	184 (77.0)	45 (18.8)	9.7 ± 5.5 (0.5, 23.5)	85 (35.76	154 (64.4)	0 (0.0)
**Total**	47 (3.3)	1,189 (82.9)	198 (13.8)	10.5 ± 6.2 (0.4, 26.8)	318 (22.2)	1,113 (77.6)	3 (0.2)

ILAA - implant-line A angle

SD – standard deviation

Table 5 shows the results for LCD, MBBH, TT, MBT0, and MBT4, in relation to each tooth. It could be observed that:

- The mean LCD was lower for CI in relation to IL and CA;
- The mean MBBH was slightly higher for CI in relation to LI, but markedly higher for CI in relation to CA:
- The mean TT was higher for CA, followed by CI and LI;
- The mean MBT0 was higher for CA, followed by LI and CI;
- The mean MBT4 was higher for CA and similar between CI and LI.

There was a statistically significant difference of MBBH, MBT0 and MBT4 mean values between the groups of male and female patients, when all teeth were considered (*p* < 0.001 and higher mean values for males for all three comparisons, Mann-Whitney test), when all measurements were considered. The mean ± SD (min, max) values for each of these distances, for male and female were, respectively:

- MBBH: 20.8 ± 3.6 (11.2, 29.5) and 18.6 ± 3.3 (8.1, 29.8);
- MBT0: 8.2 ± 1.9 (3.9, 21.2) and 7.6 ± 1.6 (3.7, 13.2);
- MBT4: 9.2 ± 2.0 (4.2, 15.9) and 8.8 ± 2.0 (2.7, 25.5).

**Table 5:** Labial concavity depth (LCD), mandible basal bone height (MBBH), tooth torque (TT), and mandibular bone thickness (MBT0, at tooth apex; MBT4, at 4 mm inferior to MBL0) in relation to each tooth

Tooth	LCD (mm)	MBBH (mm)	TT (degrees)	MBT0 (mm)	MBT4 (mm)
	mean ± SD (min, max)				
43	3.5 ± 1.6 (0.5, 9.6)	17.0 ± 2.9 (10.2, 25.7)	156.5 ± 12.0 (60.4, 179.1)	8.7 ± 1.7 (4.5, 13.2)	9.2 ± 1.8 (3.3, 13.7)
42	3.4 ± 1.4 (0.5, 8.9)	20.3 ± 3.1 (12.1, 29.8)	153.5 ± 10.0 (112.7, 179.5)	7.6 ± 1.8 (3.7, 21.2)	8.8 ± 1.9 (3.9, 15.9)
41	2.9 ± 1.4 (0.4, 8.6)	21.1 ± 3.1 (14.3, 29.5)	155.8 ± 9.8 (121.7, 179.7)	7.2 ± 1.5 (3.8, 12.3)	8.9 ± 2.4 (3.4, 25.5)
31	2.9 ± 1.4 (0.3, 9.0)	21.5 ± 3.1 (14.3, 29.5)	155.7 ± 10.0 (121.1, 196.7)	7.2 ± 1.5 (4.1, 12.1)	8.7 ± 2.0 (4.0, 14.3)
32	3.4 ± 1.5 (0.6, 10.1)	20.1 ± 3.1 (11.3, 28.6)	153.5 ± 9.8 (119.5, 178.9)	7.6 ± 1.6 (4.2, 11.6)	8.8 ± 1.9 (3.9, 15.9)
33	3.4 ± 1.6 (0.4, 10.1)	16.7 ± 2.9 (8.1, 24.3)	157.1 ± 9.7 (125.2, 179.0)	8.7 ± 1.8 (4.5, 14.1)	9.3 ± 1.9 (2.7, 14.4)
Global	3.2 ± 1.5 (0.3, 10.1)	19.5 ± 3.6 (8.1, 29.8)	155.4 ± 10.3 (60.4, 196.7)	7.8 ± 1.8 (3.7, 21.2)	9.0 ± 2.0 (2.7, 25.5)

LCD – Labial concavity depth; MBBH - Mandible basal bone height; TT - tooth torque; MBT0 - mandibular bone thickness at tooth apex; MBT4 - mandibular bone thickness at 4 mm inferior to MBL0

SD – standard deviation

Table 6 presents the results of correlation tests among the variables included in this study. There was only one very strong association, three moderate associations, and the rest were either weak or very weak associations.

**Table 6:** Correlation between several variables

Factor	Age	ILAA	LCA	LCD	MBBH	TT	MBT0	MBT4
	*r*/*r*_*s*_ (*p* value)							
Sex ^a^	-0.061 (0.021)	0.332 (< 0.001)	-0.099 (< 0.001)	0.044 (0.092)	-0.287 (< 0.001)	-0.031 (0.245)	-0.155 (< 0.001)	-0.095 (< 0.001)
Age ^b^		-0.293 (0.001)	-0.006 (0.834)	-0.022 (0.411)	0.069 (0.009)	0.166 (< 0.001)	-0.007 (0.783)	0.018 (0.498)
Tooth ^a^		-0.053 (0.575)	0.033 (0.218)	0.167 (< 0.001)	-0.536 (< 0.001)	0.047 (0.078)	0.354 (< 0.001)	0.113 (< 0.001)
ILAA ^b^			-0.333 (< 0.001)	-0.091 (0.331)	-0.035 (0.709)	-0.578 (< 0.001)	0.056 (0.552)	0.072 (0.442)
LCA ^b^				-0.220 (< 0.001)	-0.087 (< 0.001)	0.435 (< 0.001)	0.273 (< 0.001)	0.177 (< 0.001)
LCD ^b^					0.226 (< 0.001)	-0.073 (0.006)	-0.138 (< 0.001)	-0.231 (< 0.001)
MBBH ^b^						0.022 (0.401)	-0.369 (< 0.001)	-0.296 (< 0.001)
TT ^b^							-0.124 (< 0.001)	-0.158 (< 0.001)
MBT0 ^b^								0.818 (< 0.001)

LCA - Labial concavity angle; LCD – Labial concavity depth; MBBH - Mandible basal bone height; TT - tooth torque; MBT0 - mandibular bone thickness at tooth apex; MBT4 - mandibular bone thickness at 4 mm inferior to MBL0

^a^ Spearman correlation

^b^ Pearson correlation

Very strong association: *r* > 0.850

Strong association: *r* = 0.650 - 0.850

Moderate association: *r* = 0.350 - 0.649

Weak association: *r* = 0.200 - 0.349

Very weak association: *r* < 0.200

### Logistic regression

Patients’ sex, tooth region, LCA, LCD, MBBH, MBT0, and MBT4 were the factors identified by the univariate binary logistic regressions to possibly have an influence on the occurrence of unsafe implant placement, with tooth region (CI in relation to IL and CA), LCA (decrease of the angle), MBBH (decrease), and MBT0 (decrease) remaining statistically significant in the multivariable model (Table 7).

**Table 7:** Univariate and multivariable binary logistic regressions for cortical bone perforation or invasion of the 2 mm secure distance from the surrounding anatomical structures (in relation to no perforation), for bone-driven implant position

Factor	Univariate			Multivariable	
	OR (95% CI)	*p* value		OR (95% CI)	*p* value
*Sex* Male					
	1			1	
Female	2.633 (2.040, 3.398)	< 0.001		1.147 (0.767, 1.715)	0.504
*Age*	1				
Increase by 1 year	0.997 (0.989, 1.006)	0.558		-	
*Tooth region*					
Cental incisor	1			1	
Lateral incisor	0.277 (0.187, 0.413)	< 0.001		0.161 (0.093, 0.277)	< 0.001
Canine	0.152 (0.104, 0.223)	< 0.001		0.136 (0.069, 0.267)	< 0.001
*LCA*	1			1	
Increase by 1 degree	0.916 (0.900, 0.933)	< 0.001		0.943 (0.920, 0.966)	< 0.001
*LCD*	1			1	
Increase by 1 mm	1.150 (1.052, 1.256)	0.002		1.122 (0.984, 1.278)	0.085
*MBBH*	1			1	
Increase by 1 mm	1.086 (1.048, 1.125)	< 0.001		0.798 (0.741, 0.860)	< 0.001
*TT*	1				
Increase by 1 degree	1.009 (0.997, 1.021)	0.128		-	
*MBT0*	1			1	
Increase by 1 mm	0.284 (0.246, 0.328)	< 0.001		0.255 (0.192, 0.338)	< 0.001
*MBT4*	1			1	
Increase by 1 mm	0.502 (0.459, 0.550)	< 0.001		1.011 (0.808, 1.264)	0.924

OR – odds ratio; 95% CI – 95% confidence interval; LCA - Labial concavity angle; LCD – Labial concavity depth; MBBH - Mandible basal bone height; TT - tooth torque; MBT0 - mandibular bone thickness at tooth apex; MBT4 - mandibular bone thickness at 4 mm inferior to MBL0

### Lingual foramina

The lingual foramen was observed in 224 exams, 93.7% of the cases. In two CBCT exams two lingual foramina were identified. In the great majority of the cases (*n* = 143, 63.3%), the foramen was located in the midline. In other exams, the foramen was observed located apically to tooth 41 (*n* = 42, 18.6%), to tooth 31 (*n* = 38, 16.8%), one case each (0.4%) located apically to tooth 32, to tooth 33, and between teeth 41 and 42.

## Discussion

The main aim of this study was to evaluate the potential for unsafe implant placement during virtual CBCT IIP planning in the anterior mandible. Based on the present findings, it can be asserted that the vast majority of the alveolar sockets in this region was not suitable for IIP. The frequency of perforation was significantly higher for implants placed in the prosthetically driven position compared to implants placed in the bone-driven position. The null hypothesis was thus rejected. These results are in agreement with those observed in a similar study but focused on the anterior maxilla [9]. Some factors were associated with unsafe implant placement, according to the logistic regression, namely tooth region, LCA, MBBH, and MBT0.

One of them was the tooth region. Central incisors had the highest risk of unsafe placement compared to the other tooth regions, namely, 86.4% higher than canines and 83.9% higher than lateral incisors. This is greatly related to the limited volume of bone in the incisor region of the mandible [17, 18], and the anatomical particularities of the region may be the main reason for a high rate of unsafe implant placement even in a bone-driven position, 77.8%, in comparison to when IIP are planned in the anterior maxilla, 58.2%, according to a previous study [9]. These results contradict Tsai et al. [15], which observed that the canine region had the highest prevalence of labial bone perforation, followed by the central incisor, and lastly, the lateral incisor region. This discrepancy is likely due to the fact that Tsai et al. [15] had a virtual implant diameter of 4.3 mm in the canine region, whereas 3.75 mm was set for the present study. Moreover, they only investigated perforation of the labial bone and did not include the lingual bone. Our results showing incisors as more prone to perforation than canines, is likely due to the lesser amount of available bone in the region, corresponding to a lesser mandibular bone thickness [19].

Another factor associated with unsafe implant placement was LCA. The probability of perforation was reduced by 5.7% for every 1-degree increase in the LCA. In agreement, Tsai et al. [15] observed that the mean LCA was smaller for the sites in which perforation of the labial cortical bone occurred, in comparison to the non-perforation sites. A smaller LCA will generate a deeper buccal concavity, increasing the chance of insufficient bone volume for an implant. With less bone volume available in the buccal-lingual dimension at the tip of the angle, the possibility to reposition the implant without perforation becomes more limited. The mean LCA was approximately 146 degrees, although a relatively large range of nearly 80 degrees between maximum and minimum value was noted between the subjects.

There was a moderate correlation between the tooth region and MBBH. The mean MBBH was greater for the central incisors compared to the other regions. There was only a slight difference between the central and lateral incisors, whereas the canines had a markedly lower mean value, the same observed in other study [16]. This observation corresponds to an inverse relationship with the mean minimum implant length, where the central incisors had the shortest, followed by the lateral incisors, and lastly the canines. This aligns with the anatomy of the anterior mandibular teeth, where the canines have the longest roots and therefore are placed deeper into the basal bone, therefore needing a longer implant in order to anchorage the implant with a minimum of 4 mm of basal bone, which consequently results in a decrease in the available MBBH.

Shorter MBT0 was associated with higher risk of unsafe implant placement, in agreement with another study [15]. The mean MBT0 was at its greatest in the canine region, followed by lateral, and lastly central incisors, similar to the results of other study [17]. There was a very strong correlation between MBT0 and MBT4, where MBT0 < MBT4 in the different regions. When considering the anatomy of the mandibular bone, this is expected, as the mandible is at its narrowest at the alveolar crest and generally increases in width towards the basal bone, with some slight anatomical variations [15, 16]. This means that MBT0 can be generally seen as the “bottle neck” region from the buccal-lingual point of view – if there will be enough buccal-lingual distance to safely accommodate an implant, then surely there will be space at MBT4, the minimum distance at which the implant is expected to apically be placed in order to achieve primary stability and minimize the risk of early implant loss. As it can been seen from the results of the regression analysis MBT0 was associated with unsafe implant placement, but not MBT4.

Most of the implants virtually planned to be placed in the prosthetically-driven position either perforated an adjacent cortical bone (13.8%) or invaded the minimum-security distance of 2 mm from adjacent structures (82.9%). Therefore, an inclination of the implant from the long axis of the tooth was needed for the great majority. This implies that implants that are planned to be immediately placed in the anterior mandibular area will, in most cases, need to have its coronal part tilted buccally in order to get enough anchorage of the available bone apical to the alveolar socket. The same was observed in other study, but for the anterior maxillary area [9]. From the prosthetic point of view, this would mean that implant-supported single crowns in the anterior mandible would need to be cemented on a custom-made prosthetic abutment, with this having a mean buccal-palatal angulation of 10 degrees, reflecting the mean ILAA (10.5 ± 6.2 degrees), although higher – around 15 degrees - for the central incisors. A prosthetic alternative for this issue would be the use of individualized abutments with an angled screw channel [20], making it possible to restore the implant with a screw-retained crown instead. However, the results of a systematic review suggested that differences in angulation of dental implants in the mandible might not affect the implant survival [21].

Furthermore, there was a moderate correlation between ILAA and TT, where a smaller ILAA results in a bigger TT. This is due to the fact that the closer the TT comes to 180 degrees, the less angulation is needed to reach the bone position with the maximum bone volume. A bigger TT implies a tooth more perpendicular to the mandibular bone. The ILAA is the difference in angulation between the prosthetically driven position and the bone-driven position. A small ILAA therefore indicates that the tooth is already close to an optimal angulation when repositioned in the bone-driven position, minimizing the risk of an unsafe implant placement. A smaller TT indicates a tooth in need of a greater angular correction to reach the bone-driven position, resulting in a greater ILAA.

Primary stability has been considered as an important factor for dental implant treatment [22], probably being even more crucial when an implant is planned to be placed in an extraction socket. It is often true that the extraction socket is broader than the implant, and therefore the implant will mostly, or even only, be able to be anchored in the apical part of the socket. The primary implant stability can then be compromised [23]. One of the assumed crucial factors in order to obtain primary stability when implementing IIP, is a minimum of 4 mm of apical anchorage [12, 13]. Here is also important to call attention to the quality of bone where the implant will be anchored, since sites with poorer bone quality may statistically affect implant failure rates in a negative way [24]. With all this in mind, a longer implant can be necessary when performing IIP than when the implant is planned to be placed in a healed or pristine bone site. According to the present study, the minimum value of implant length in bone-driven position ranged from 9.5 to 21.0 mm in sockets of the anterior mandible. The considerable difference in length between the minimum and maximum values implied that there could be a large variation between subjects, meaning the required implant length was highly individual. It is therefore necessary to examine every individual separately.

The limitations of the present study include the fact that the validity of these results relies on the accuracy of CBCT images. Moreover, the measurements were based on single implant placement only, meaning the required distance between implants when more than one implant was placed adjacently was not taken into consideration. In addition, despite the meticulous initial interexaminer calibration, there is a possibility of inconsistencies arising in the data collected by study participants while individually reviewing the tomographic images. One additional limitation to consider is the distance LCD, which was considered as one of the variables here due to its investigation in a previous study with similar aims [15]. Although there is a standardized orientation for the head during CBCT examination, small inclinations of the head cannot be ruled out, which could have some influence on the calibration of the LCD distance among different individuals.

Perforation of the cortical bone wall can be greatly minimized when the implant is placed in a bone-driven position compared to a prosthetically-driven position. It is important to pre-operatively evaluate the morphological features of the implant site for risk assessment and to individualize the treatment plan. Two-dimensional exams such as orthopantomograms are usually more accessible, are less costly, and emit low radiation doses. However, they present limited information concerning pre-assessment of the risk of cortical bone perforation. Exams such as CBCT provide an accurate three-dimensional perception of the surrounding anatomic structures [25, 26], which is essential in planning IIP in a region with a high risk of unsafe placement such as the anterior mandibular region.

## Conclusions

1. The possibility of safely placing immediate implants in the anterior mandible is significantly higher for bone-driven position in comparison to prosthetically driven position (22.2% vs. 3.3%, respectively);
2. The minimal implant length possible without cortical bone perforation while respecting a secure distance from adjacent anatomical structures and a minimum apical anchorage of 4 mm is greatest for canines and shortest for central incisors;
3. The general mean ILAA is approximately 10^o^, though higher for central incisors, i.e., 15^o^;
4. Tooth region (CI in relation to IL and CA), LCA (decrease of the angle), MBBH (decrease), and MBT0 (decrease) are associated with an unsafe IIP in the anterior mandible;
5. The sex of the patient might be worth considering, with regards to the anatomical variations in favor of men;
6. The position and angulation of the individual tooth in comparison to the basal bone needs to be considered separately during implant placement planning, especially in the incisal region.

## Data Availability

The datasets generated and/or analyzed during the current study are not publicly available due the General Data Protection Regulation (EU) 2016/679, but are available from the corresponding author on reasonable request.
